# Towards the use of diffuse reflectance spectroscopy for real-time in vivo detection of breast cancer during surgery

**DOI:** 10.1186/s12967-018-1747-5

**Published:** 2018-12-19

**Authors:** Lisanne L. de Boer, Torre M. Bydlon, Frederieke van Duijnhoven, Marie-Jeanne T. F. D. Vranken Peeters, Claudette E. Loo, Gonneke A. O. Winter-Warnars, Joyce Sanders, Henricus J. C. M. Sterenborg, Benno H. W. Hendriks, Theo J. M. Ruers

**Affiliations:** 1 Department of Surgery, the Netherlands Cancer Institute–Antoni van Leeuwenhoek, Plesmanlaan 121, Postbus 90203, 1066 CX Amsterdam, The Netherlands; 2 In-body Systems, Philips Research, High Tech, Campus 34, 5656 AE Eindhoven, The Netherlands; 3 Department of Radiology, the Netherlands Cancer Institute–Antoni van Leeuwenhoek, Plesmanlaan 121, 1066 CX Amsterdam, The Netherlands; 4 Department of Pathology, the Netherlands Cancer Institute–Antoni van Leeuwenhoek, Plesmanlaan 121, 1066 CX Amsterdam, The Netherlands; 5 Biomedical Engineering and Physics, Amsterdam UMC, University of Amsterdam, Meibergdreef 9, 1105 AZ Amsterdam, The Netherlands; 6 Biomechanical Engineering, Delft University of Technology, Mekelweg 5, 2628 CD Delft, The Netherlands; 7 Technical Medical Centre, University of Twente, Drienerlolaan 5, 7522 NB Enschede, The Netherlands

**Keywords:** Breast cancer surgery, Intraoperative margin assessment, Optical technology, Real-time

## Abstract

**Background:**

Breast cancer surgeons struggle with differentiating healthy tissue from cancer at the resection margin during surgery. We report on the feasibility of using diffuse reflectance spectroscopy (DRS) for real-time in vivo tissue characterization.

**Methods:**

Evaluating feasibility of the technology requires a setting in which measurements, imaging and pathology have the best possible correlation. For this purpose an optical biopsy needle was used that had integrated optical fibers at the tip of the needle. This approach enabled the best possible correlation between optical measurement volume and tissue histology. With this optical biopsy needle we acquired real-time DRS data of normal tissue and tumor tissue in 27 patients that underwent an ultrasound guided breast biopsy procedure. Five additional patients were measured in continuous mode in which we obtained DRS measurements along the entire biopsy needle trajectory. We developed and compared three different support vector machine based classification models to classify the DRS measurements.

**Results:**

With DRS malignant tissue could be discriminated from healthy tissue. The classification model that was based on eight selected wavelengths had the highest accuracy and Matthews Correlation Coefficient (MCC) of 0.93 and 0.87, respectively. In three patients that were measured in continuous mode and had malignant tissue in their biopsy specimen, a clear transition was seen in the classified DRS measurements going from healthy tissue to tumor tissue. This transition was not seen in the other two continuously measured patients that had benign tissue in their biopsy specimen.

**Conclusions:**

It was concluded that DRS is feasible for integration in a surgical tool that could assist the breast surgeon in detecting positive resection margins during breast surgery.

*Trail registration* NIH US National Library of Medicine–clinicaltrails.gov, NCT01730365. Registered: 10/04/2012 https://clinicaltrials.gov/ct2/show/study/NCT01730365

## Background

The current primary treatment of breast cancer includes a multimodal approach with a combination of surgery and radiotherapy, and depending on the subtype and the extent of the disease, systemic treatment. Optimal surgical treatment is achieved when all tumor tissue is resected, and thus histopathological evaluation of the resection specimen reveals no tumor positive margins. However, resecting too much healthy tissue compromises cosmetic outcome. Since tumor positive resection margins are associated with a higher recurrence rate, these patients require additional treatment with boost radiotherapy or re-excision surgery [1, 2]. Along with the impact of additional treatment on healthcare budgets, both boost radiotherapy and secondary surgery impair cosmetic outcomes [3], increase morbidity [4, 5] and can affect quality of life [6–8]. The surgeon is thus balancing between completely resecting the tumor and, sparing as much healthy tissue as possible [9]. Performing surgery while doing justice to both is difficult since visually recognizing tumor tissue on the surgical margin is extremely challenging. In addition, all currently available intra-operative margin assessment techniques have their own pitfalls, such as reduced sensitivity, requirement of skilled personnel, are labor-intensive, and can be operator dependent [10–12]. Therefore, breast surgeons are in need of a robust margin assessment tool that can assist them real-time in defining the optimal resection plane to ensure clear margins, and will also help them to minimize the resected specimen volume [13–18].

Diffuse reflectance spectroscopy (DRS), a light-based technology, has shown promising results for discriminating normal breast tissue from tumor tissue and may address the need for a margin assessment device [19–22]. The principle behind DRS is that light interacts with tissue through scattering and absorption. The absorption is related to the chemical composition of the tissue whereas the scattering is related to the subcellular morphology. The reflected light, detected after tissue interaction, has an altered spectrum compared to the incoming light. Thus, the diffuse reflectance spectrum represents aspects of the composition and subcellular morphology of the measured tissue. Ultimately, incorporating DRS technology in instruments, such as a surgical knife, could potentially provide the surgeon with additional information that reflects the histopathology of the tissue at the resection margin. The surgeon can use this information as guidance to determining the optimal resection place for excising a breast tumor.

There have been some publications on the feasibility of DRS for breast biopsy and surgery applications [23–26]. However, many of these publications struggled with correlating the exact tissue volume measured by DRS in vivo to the proper location in the histopathology slides processed post-operatively. This is an important factor as the ‘gold standard’ for the evaluation of surgical margins is microscopic assessment by a pathologist. Thus, a mismatch between the optical measurements and the histopathology hampers the development of robust classification algorithms and validation of the technology. For the purpose of developing a reliable database of DRS measurements we developed a special biopsy needle with embedded optical fibers [27]. This tool enables DRS measurements and subsequent biopsy of the same tissue volume as measured spectroscopically. Although it is tempting to see this study as an attempt to perform spectroscopy guided biopsy, this was explicitly not the purpose of this study. The robust dataset gathered in this setting can be used for developing classification models and validating DRS technology for tissue characterization. The paper describes how we developed and tested three predictive classification models, based on different types of input data, to accurately classify the DRS measurements. Furthermore, we investigated the feasibility of acquiring in vivo DRS measurements and classifying these. To this end, DRS measurements were performed continuously along an entire needle trajectory during ultrasound guided breast biopsy procedures, and classified based on a classification model.

## Methods

### Study design

Patients suspected of having breast cancer (after palpation, X-ray and US-imaging) that required diagnostic biopsies were asked to participate in this observational study that was approved by the Institutional Review Board of the Netherlands Cancer Institute. Written informed consent was obtained from all patients prior to the biopsy procedure. Patients with suspected sensitivity to light (e.g. patient who have had photodynamic therapy) were excluded, as well as patients that had received prior chemotherapy, endocrine therapy or radiation therapy recently (i.e. within 5 years). Patients were also excluded with breast implants and those that needed a stereotactic breast biopsy. In all patients, a biopsy was obtained after the last measurement in tumor tissue. The biopsy specimen was colored with pathology ink on the distal side to indicate which side had been in contact with the fibers during measurements. The biopsy specimens were used for diagnostic assessment and to confirm histopathology of the tumor measurement location by evaluating the first 2 mm of the side of the biopsy specimen that had been in contact with the optical fibers. Patients were only included when the pathology results of the lesion (including the other diagnostic biopsy specimens) indicated the lesion was an invasive breast tumor.

### Spectrometers and optical biopsy needle

**Fig. 1:**
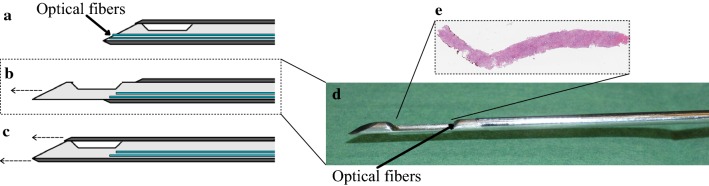
Biopsy needle with integrated optical fibers. **a** In the initial phase the tissue is in contact with the fibers. **b** When the release button is pressed the cutting mechanism extends forward while the fibers retract. The tissue can now enter the biopsy cavity. **c** When pressing the release button further the outer stylet will extend forward, thereby cutting the tissue in the biopsy cavity from its surrounding. **d** Photograph of the biopsy needle with extended inner stylet, with the cutting mechanism protruded similar to situation **b**. **e** Example of an H&E stained slide of the biopsy specimen. The side of the specimen that was not in contact with the fibers (in this case the left side) is marked with red pathology ink directly after retrieving the biopsy specimen from the cavity

DRS measurements were obtained with a specially designed optical biopsy needle (Fig. 1). This optical biopsy needle, which had integrated optical fibers, combined the ability to measure DRS spectra with biopsy functionality [27]. The 14G optical biopsy needle had one 100 μm fiber for illumination and two 200 μm fibers for collecting the light (Invivo); with a 20 mm cavity for the tissue biopsy. The collecting fibers were placed next to each other, and the distance between the illuminating and collecting fibers was 1.36 mm, which resulted in a penetration depth of approximately 1–2 mm. The optical biopsy needle was attached to two spectrometers that resolved light in the visual wavelength range (DU420A-BRDD, Andor Technology) and the NIR wavelength range (DU492A-1.7, Andor Technology). After measuring, the spectra of the two spectrometers were stitched together, to form a continuous spectrum between 400 and 1600 nm [28]. In the full spectrum and selected wavelengths classification models the first 100 nm was removed since this wavelength range was highly affected by noise which may influence the machine learning algorithms that were used for the development of the models.

### DRS point measurements

In 27 patients, measurements were acquired in a point-based manner, thus the measurement needle was first held still in normal tissue, and subsequently in the breast tumor where a biopsy was taken. At each measurement location, three DRS measurements (10 spectra per measurement) were acquired and averaged. Performing a single measurement took approximately 10 s. In this measurement time a total of 30 high quality DRS spectra were obtained over the full wavelength range which is necessary for building a database that can be used for classification model development. During the time the DRS measurements were obtained an US-image was made with the needle tip in view. These US-images were evaluated by a radiologist to confirm correct positioning of the needle in either normal breast tissue or tumor tissue.

### DRS continuous measurements

In five additional patients, DRS measurements were obtained in a continuous mode. Here the measurements were obtained along the entire needle trajectory starting in the normal tissue, continuing through the transition zone of normal-tumor, and ending in the tumor. To enable real-time acquisition of the data adjustments were made to the settings to increase the acquisition rate. The framerate in continuous mode was approximately one spectrum per second. The integration time was set to 0.35 s for all measurements along the needle trajectory. Prior to measuring the clocks of the US device and the laptop controlling the DRS set-up were synchronized thus allowing the US-images to be registered to the DRS measurements. At one location at a distance from the tumor, and at the final measurement location (also the location of the biopsy specimen) the needle was kept still to ensure sufficient data of both healthy tissue and tumor tissue (similar to the point measurements). At these locations, 10 DRS measurements were acquired, as well as an US-image.

### Pre-processing point measurements

At each point measurement location three spectra were obtained which were averaged to calculate a mean spectrum of each measurement location. Subsequently all spectra were normalized with the standard normal variate (SNV) method [29]. Outlier detection was performed to ensure that the classification models were developed with reliable data [30]. A cut-off of 3 times the standard deviation was chosen as threshold.

### Development of classification models

For development of the first classification models only the point-based measurements were used as for these measurement US images and histopathology provided information on the nature of the tissue in front of the needle during a measurement. For the continuous data this information was not available for each measurement in the trajectory.

**Fig. 2:**
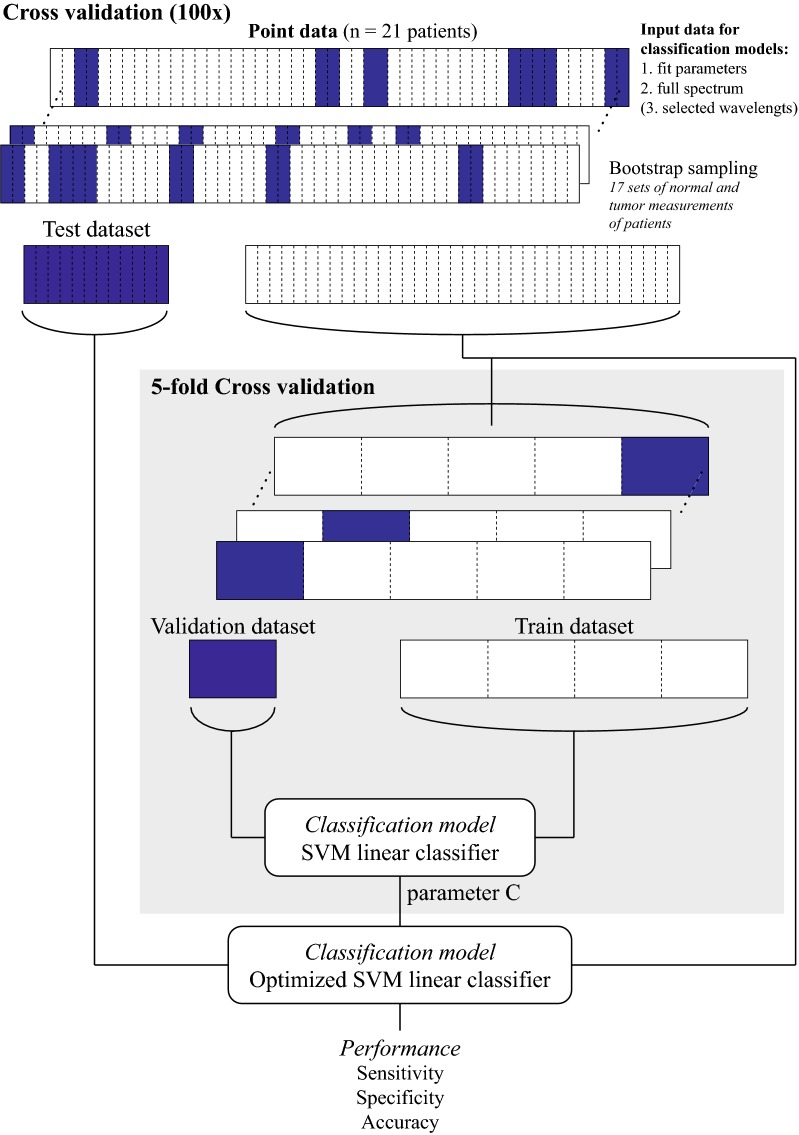
Schematic overview of training and testing of the classification model. In the inner loop the SVM is optimized (fivefold cross validation), this optimized model is subsequently used with the test dataset. The outer loop is performed 100 times. The sensitivity, specificity and accuracy are averaged over all iterations

To ensure a balanced dataset, only patients which had both the normal and tumor measurement available after outlier detection were used. All classification models were constructed with perClass (Academic version 5.0, PR Sys design) in Matlab (2015a, the MathWorks). Figure 2 depicts a schematic flowchart of the approach to build the classification models.

### Fit parameter classification models

The input for the first classification model was optical fit parameter data. To calculate these fit parameters from the measured DRS spectra, the measurements were quantified using an analytical fit model based on diffusion theory. This can be considered as a feature reduction method in which the measured spectra is translated into chemically or physiologically meaningful parameters [31]. In order to do so, the fit model required the absorption spectra of substances present in tissue, including: blood, fat, water, β-carotene, collagen, and bilirubin. The fit then optimized the parameters in such a way that the modelled spectrum matched the measured spectrum. The optical fit parameters generated by the fit model were: amount of blood (%), oxygen saturation (StO_2_), total amount of fat plus water, fraction of fat, scattering at 800 nm, α and *b* (from the formula describing the reduced scattering, $\mu^{\prime}_{s} = \alpha \lambda^{ - b}$) amount of bilirubin, fraction of Mie scattering (in relation to the total scattering), amount of β-Carotene, and amount of collagen. The amount of water was calculated from the optical parameters describing the total amount of fat and water and the fat fraction. The amount of water together with the amount of fat allowed deriving the ratio between fat and water for each measurement location [21, 28].

Different combinations of fit parameters were used as input for this classification model. To limit the required computational effort, these combinations were formed by combining fit parameters that had shown the ability to discriminate between normal and tumor tissue previously, i.e. blood, StO_2_, scattering at 800 nm, fraction Mie scattering, β-carotene, collagen and, the ratio between fat and water (F/W-ratio). With these seven fit parameters combinations (*c*) were made that consisted of either one or multiple fit parameters with one combination including all seven fit parameters (*c* = 127, $c = \sum\nolimits_{k = 1}^{7} {\frac{n!}{((n - k)!k!)}}$) with *n* the number of fit parameters to choose from, and *k* the amount of elements in the combination). As the F/W-ratio previously proved to be an excellent discriminator [21, 32], only fit parameter combinations that included the F/W-ratio were used as input for the first classification model. The final set of combinations consisted of 64 possibilities.

### Full spectrum classification model

The input for the second classification model was the full wavelength spectrum without any feature reduction. Thus, each wavelength between 500 and 1600 nm (i.e. the full spectrum) was used as input to the model, resulting in 1100 features for the classification model.

### Selected wavelengths classification model

The input for the third classification model consisted of a limited number of wavelengths. The selection of these wavelengths was based on the results of a two-sided Wilcoxon rank sum test (alpha = 0.05). All normal and all tumor measurements were used in the test. For each wavelength the Wilcoxon rank sum test assess whether two samples of observations (in this case the normal measurements and the tumor measurements) are from the same distribution. This statistical test was used to identify wavelength regions that were significantly different between the normal and tumor spectra (mean *p* value below 0.05). In each of these regions, the wavelength with the lowest p-value was selected as a wavelength for the selected wavelengths model.

### Classifier

A linear support vector machine (SVM) formed the classifier in the classification model [33, 34]. This machine learning technique constructed an optimal separating linear hyperplane between two classes in a higher dimensional space by creating the biggest margin between measurements of two classes.

### Bootstrap sampling and cross-validation

To avoid selection bias, a bootstrapping technique was used to randomly select 17 subsets of (not necessarily different) patients as training data for model development. On average, both the normal and tumor measurements of 11.9 (± 1.3) unique patients were used as training data in each of the 100 iterations. The remaining patients that were not selected for training were used for testing the model. Subsequently the SVM was optimized with a fivefold cross validation of the training data to find the optimum for the regularization parameter C. The unseen test data was then classified with the optimized SVM model. This process was performed a hundred times in order to test generalizability of the classification model. Measurements provided to the classification model were classified as either ‘normal’ or ‘tumor’. With this binary output the sensitivity, specificity, and accuracy in each bootstrap iteration was calculated for discriminating normal measurements from tumor measurements. By averaging these model performance parameters over the 100 iterations the mean sensitivity, mean specificity, mean accuracy, and mean Matthews Correlation Coefficient (MCC) were determined, and these were used to compare the performance of different classification models. Besides the binary output, the classification models could also generate the probability of a measurement being either ‘normal’ or ‘tumor’.

### Classification of continuous data

**Fig. 3:**
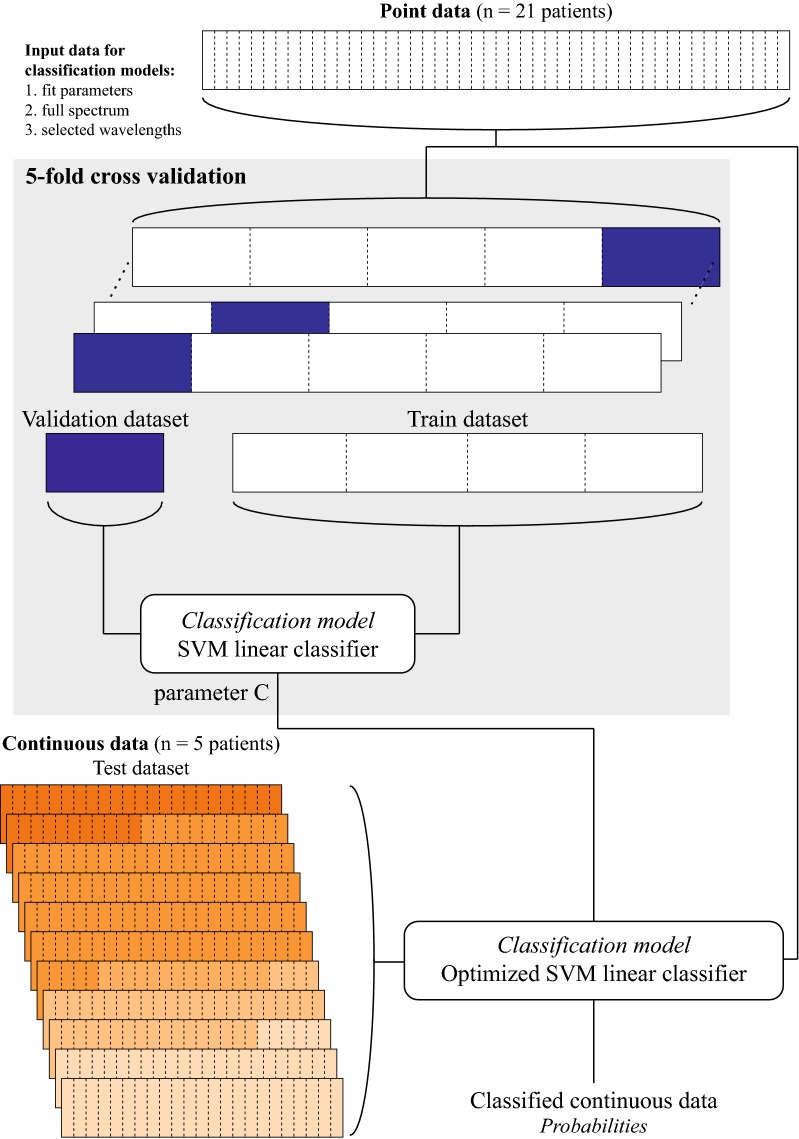
Schematic overview of the classification model development with point measurements to classify continuous measurements. Again, either the fit parameters data, full spectrum data or, selected wavelengths data is used as input for the classification model development

The continuous data was preprocessed, by normalizing it using the SNV method. Spectra were not averaged and no outlier detection was performed. Along the needle trajectory, US-images were captured of the biopsy needle while it was positioned in healthy tissue during the first measurement, and when the needle was at the final measurement location, targeting to be in the tumor. For the classification of the continuous data, all point measurements were used to build another three classification models with the different input data (i.e. fit parameter, full spectrum, and selected wavelengths) (Fig. 3). No measurements of the continuously acquired data were used in the development of any of the models, they were only provided to the classification model to be classified. Therefore these needle trajectory measurements were not labeled based on US-imaging or pathology.

## Results

In total 32 patients were measured and had unambiguous pathology results. Of these patients, 27 formed the point measurement dataset and 5 the continuous dataset. From the 27 point-based measurement patients, in one patient the biopsy specimen was absent and two patients had biopsy specimens that were clearly damaged during processing of the tissue. In these three cases careful evaluation of the US images by a radiologist revealed that the needle tip was certainly placed a few millimeters inside the tumor and therefore these patients were still included in the analysis. Four patients were excluded from the analysis because (1) the side of the biopsy specimen that had been in contact with the fibers during the measurement consisted of healthy tissue over the extent of a few millimeters and (2) according to the radiologist the needle was moved between the measurement and biopsy. No patients were excluded because the tumor was too close to the skin, thus prohibiting the acquisition of measurements of healthy tissue.

In the procedure of outlier detection, two measurement locations were detected. An explanation for the first outlier might be that the needle tip was in a pool of blood during the normal measurements, which was confirmed by the high blood content according to the fit parameters. As for the second outlier, the histopathology of this measurement location showed benign tissue in the biopsy specimen. The patients to which these locations belonged were also excluded to ensure a balanced dataset.

Thus, in total 6 patients were excluded from the point measurement dataset. The remaining 21 patients, in whom point measurements were obtained, were included for further analysis. The patient characteristics of both patient datasets seem similar and are summarized in Table 1.

**Table 1:** Patient characteristics of point measurements dataset and continuous measurements dataset

Patient characteristics	Point measurements (n = 21)	Continuous measurements (n = 5)
Mean age (std)	53.4 year (12.1)	60.6 year (10.2)
Menopausal status		
Premenopausal	8	1
Perimenopausal	2	0
Postmenopausal	10	4
Unknown	1	0
Mean tumor size (US imaging) (std)	25.5 mm (11.1)	37.2 mm (25.1)
Cancer type^a^		
Invasive ductal carcinoma	19	3
Invasive lobular carcinoma	2	1
Mucinous adenocarcinoma	0	1

^a^The histopathology was based on the histopathology of all biopsy specimens taken in that patient

### Classification models based on fit parameters

In total 64 classification models that were based on combinations of fit parameters were built. The two fit parameter combinations that generated the two classification models with the highest accuracies are listed in Table 2. The fit parameter combination of F/W-ratio and collagen was the combination that resulted in the classification model with the best performance, with a mean accuracy, sensitivity, specificity and MCC of 0.85 (0.16), 0.72 (0.33), 0.99 (0.03), and 0.74 (0.30), respectively. The second best performing classification model was based on the F/W-ratio alone, which had a slightly lower sensitivity compared with the combination of the F/W-ratio and collagen.

**Table 2:** Performance (mean accuracy, sensitivity, specificity and MCC with standard deviations) of classification models

Type of data used as input for the model	Mean accuracy (std)	Mean sensitivity (std)	Mean specificity (std)	Mean MCC (std)
Fit parameters				
F/W-ratio and collagen	0.85 (0.16)	0.72 (0.33)	0.99 (0.03)	0.74 (0.30)
F/W-ratio	0.85 (0.16)	0.71 (0.34)	0.99 (0.04)	0.72 (0.31)
Full spectrum	0.92 (0.06)	0.94 (0.10)	0.89 (0.11)	0.84 (0.12)
Selected wavelengths	0.93 (0.06)	0.95 (0.07)	0.91 (0.14)	0.87 (0.11)

### Classification model based on full spectrum

The mean accuracy, sensitivity, specificity, and MCC of the model based on the full spectrum were 0.92 (0.06), 0.94 (0.10), 0.89 (0.11), and 0.84 (0.12), respectively (Table 2). Compared to the fit parameter model, the full spectrum model had a better accuracy, sensitivity, and MCC, whereas the specificity of the fit parameter model was better. This indicates that the full spectrum classification model is useful for detecting all tumor tissue at the cost of classifying some normal tissue as tumor. With the fit parameter classification model, less normal tissue will be incorrectly classified as tumor, but also, less tumor tissue will be detected.

### Classification model based on selected wavelengths

**Fig. 4:**
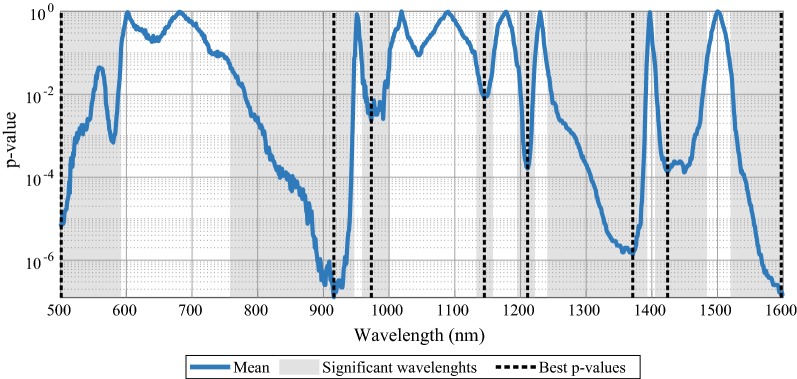
P-values of Wilcoxon rank sum test. Results of a two sided Wilcoxon rank sum test (alpha = 0.05) for each wavelength between normal and tumor measurements. The grey wavelength ranges indicate that over these wavelengths there is a significant difference between normal and tumor. The vertical dashed lines represent the wavelengths with the lowest p-value in each grey area. The eight selected wavelengths were: 501 nm, 916 nm, 973 nm, 1145 nm, 1211 nm, 1371 nm, 1424 nm, and 1597 nm

A third classification model was developed using a selection of wavelengths that were significantly different between normal and tumor spectra according to the Wilcoxon rank sum test (alpha = 0.05). Figure 4 shows the results of the Wilcoxon rank sum test. The grey parts of the graph represent wavelength areas in which the p-value was lower than 0.05. From these areas the wavelength with the lowest p-value was selected for the selected wavelengths model (vertical dashed lines). The selected wavelengths were: 501 nm, 916 nm, 973 nm, 1145 nm, 1211 nm, 1371 nm, 1424 nm, and 1597 nm.

The classification model based on these wavelengths was tested similarly to the models based on the fit parameters and the full spectrum. The mean accuracy, sensitivity, specificity, and MCC of this model was 0.93 (0.06), 0.95 (0.07), 0.91 (0.14), and 0.87 (0.11), respectively (Table 2). Compared to the fit parameter model this model has improved mean sensitivity, but reduced specificity. Despite the decrease in mean specificity, the mean accuracy and MCC of the selected wavelengths model is higher in comparison to the fit parameter model.

The classification model after feature selection also outperforms the full spectrum model as the mean accuracy, sensitivity and specificity are slightly higher. The MCC of the classification model based on a selection of wavelengths was the highest with the lowest standard deviation compared to the other models.

To ensure the improvement of model performance was related to the actual wavelengths in the set of selected wavelengths, the model performance was compared to the model performance of a subset of wavelengths that had the maximum p-value from wavelength ranges with p-values of > 0.5. The selected wavelengths for this model were: 602 nm, 681 nm, 951 nm, 1018 nm, 1095 nm, 1174 nm, 1230 nm, 1397 nm, and 1503 nm. As for the model with these eight selected wavelengths the mean accuracy, mean sensitivity, and mean specificity was 0.49 (0.12), 0.48 (0.25), and 0.50 (0.27), respectively. The MCC also displayed weak performance of this model with a mean value of 0.61 and a standard deviation of 0.32.

### Classifying continuous data

**Fig. 5:**
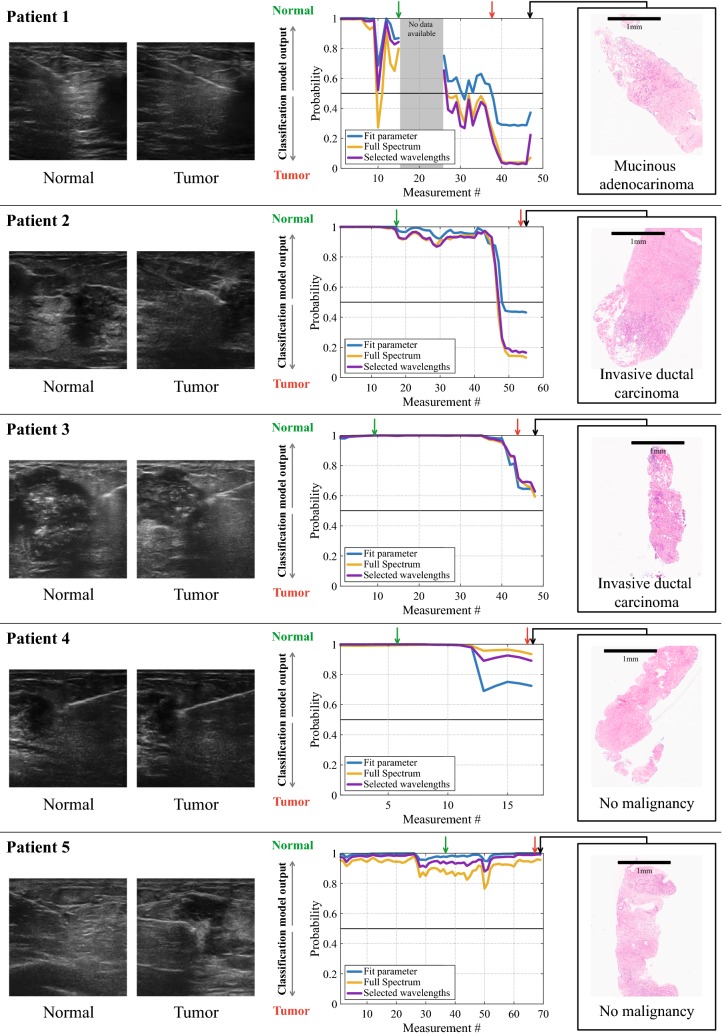
Classification of continuous data. In the left part of the image, US images taken along the needle trajectory at ‘normal’ and ‘tumor’. The middle of the image includes the outcomes of the classification algorithms, where the x-axis is the measurement number (≠ distance) and the y-axis is the probability of a measurement being normal (> 0.5) or tumor (< 0.5). The green and red arrows indicate the locations where the needle was kept still. The histopathology of the part of the biopsy specimen that was in contact with the needle is displayed in the right side

The data from the five patients that were measured in continuous mode were tested on the three classification models (fit parameters, full spectrum and selected wavelengths). To make these classification models consistent with the previous models, the fit parameter model was developed with the same fit parameters as in the previous model (F/W-ratio & Collagen) and similarly for the selected wavelengths model the same eight wavelengths (501 nm, 916 nm, 973 nm, 1145 nm, 1211 nm, 1371 nm, 1424 nm, and 1597 nm) were used. The results of the classification of the continuous data are represented in Fig. 5.

In the left part of the figure for each patient the US images at two locations along the needle trajectory (‘normal’ and ‘tumor’) are shown. The histopathology of the part of the biopsy specimen that was in touch with the fibers at the last measurement location is displayed in the right side of the figure. The black bars represent a distance of 1 mm in the histopathology image. The graphs in the center of the figure show the output of the classification models in terms of probabilities for each measurement. A probability of > 0.5 indicates a measurement is classified as ‘normal breast tissue’ by the model, whereas a probability of < 0.5 implies ‘tumor’. The x-axis represents the measurements in time, not in distance. In patient 1, there are some measurements missing because these were accidently not saved during the procedure.

The histopathologic evaluation by the pathologist revealed that there was tumor (‘mucinous adenocarcinoma’, or ‘invasive ductal carcinoma’) in the biopsy specimen of patient 1, 2 and 3. In the case of patient 4 and 5, the side of the biopsy specimens touching the fibers did not contain malignant tissue according to the pathologist. In all three patients that had invasive carcinoma in their biopsy specimen (patient 1, patient 2, and patient 3) there is a distinct decrease in probability visible in the classified DRS measurements taken along the trajectory from healthy tissue to tumor tissue. Furthermore, the first measurements of patient 1 and patient 2 are classified as normal tissue (probabilities close to one), and the final measurements are classified as tumor (probabilities close to zero) by all three models. In both these patients, the probability of the final measurement of the trajectory calculated by the full spectrum model and the selected wavelengths model are closer to zero than the output of the fit parameter model, indicating more certainty of the classification. Along the trajectory of patient 1, there is one outlier (measurement #10), which displays a distinct decrease in probability for all classification models. The measurements of patient 3 are classified as normal in the beginning of the trajectory and as the needle progressed to the tumor, the probabilities, clearly and consistently over all models, decreased. However, at the end of the trajectory, none of the three models classified the final measurements as tumor, whereas according to the biopsy specimen the needle was placed in tumor tissue.

Two patients did not have tumor tissue in the first 2 mm of the biopsy specimen that was in contact with the optical fibers (patient 4 and 5). In both cases, the outcomes of the classification models classified all measurements in the needle trajectory as normal tissue. As for patient 4, the probability of the fit parameter model does show a decrease that was not seen in the output of the other two classification models. The transition from normal tissue to tumor, seen in the patients with a malignancy (patient 1, patient 2 and patient 3), is not consistently present in the classification output of all three models in patient 4 and patient 5, whom had no malignancy in their biopsy specimens.

## Discussion

A large amount of evidence from in vivo and ex vivo studies around the world has proven that DRS can be a highly powerful tool for clinical use to discriminate tissue types. However, the technology has not been integrated with a surgical tool for real-time margin assessment. With the goal of moving towards a real-time classification tool for surgical margin assessment during breast surgery, we aimed at developing a classification model to accurately predict the type of tissue in front of the DRS tool, as well as showing the feasibility of real-time use of this technology. To reach these goals a custom made optical biopsy needle was used enabling DRS measurements and histopathology to be assessed on the same tissue volume which is inevitable for developing a robust classification model. It should be noted that this research was conducted with this set-up as step towards developing a surgical tool that can guide the surgeon, rather than to improve the yield of breast biopsy procedures. This research differs from previous work as it measures DRS over a broader wavelength range extending into the near-infrared wavelengths. Furthermore, to the authors’ knowledge, it is the first publication to test the feasibility of in vivo continuous DRS data acquisition, with a frame rate of approximately one measurement per second, which is more similar to how data will be acquired in the surgical setting.

We first used the point-based measurements to determine the performance of the classification models that were based on different input data. We found that if the fit parameter data was used as input, the combination of the F/W-ratio and collagen resulted in a model with the highest accuracy and MCC (0.85 and 0.74) compared to other combinations of fit parameters. Besides the fit parameter model, two other models were developed using the full spectrum of wavelengths or a selection of wavelengths as input. The full spectrum model had a better sensitivity compared to the fit parameter model (0.94 versus 0.72), whereas the fit parameter model had a higher specificity (0.99 versus 0.89), suggesting that the full spectrum model is more suitable for detecting tumor tissue, while the fit parameter model has less misclassifications of normal tissue. Although not statistically tested, the classification model based on a subset of selected wavelengths seems to outperform the other two models with the highest accuracy and MCC (0.93 and 0.87).

We developed three classification models (fit parameters, full spectrum and selected wavelengths) based on all available point-measurements specifically to classify the continuous measurements. Importantly, none of the continuous measurements were used for development of a classification model, they only served as test data to be classified by the classification model (Fig. 3). In all five patients, the first measurements were classified as normal tissue by the classification models, this is expected considering the fact that the needle trajectory starts in normal tissue going towards the suspected tumor tissue. In patients 1, 2, 4, and 5, the classified DRS measurements of the final measurement locations are in agreement with the pathological outcome. In patient 1, however, there is a measurement (#10) in the trajectory that is classified as ‘tumor’; this appears to be a ‘false positive’ since the distance from this location to the lesion is quite far. In the surgical pathology report following lumpectomy for this patient it was noted that there was a focus of DCIS 1.5 mm from the tumor. It could be possible that this smaller lesion was in the trajectory of the needle, explaining the decrease in probability.

The output by the classification models of the final measurement location were not in accordance with histopathologic evaluation in one patient (patient 3). In this case, the outputs of the classification models show a decrease in probability, but never reach the threshold, and the final measurement location is classified as normal by all classification models. It could be possible that the histopathology evaluation of this patient has been compromised as the removal of the biopsy specimen from the needle cavity was difficult and, since the specimen was fragmented, part of it might have been left behind. Overall, in four out of five continuous mode patients, the classification models were able to discriminate tumor tissue from normal tissue, although the fit parameter model was least convincing with probabilities closer to 0.5. In three out of five patients malignant tissue was present in the biopsy specimen and in these patients a decrease in probability of the classified measurements is also seen along the needle trajectory. This decrease is absent in the other two patients that had healthy tissue in their biopsy specimens. The fact that a decrease can be detected is an important result when considering DRS as a margin assessment tool. In a way, this trajectory can be seen as a line that at some point crosses the optimal resection plane that is perpendicular to this line. Thus, being able to detect the upcoming tumor could provide the surgeon with viable information for guidance.

A limitation of the continuous measurements is that a biopsy was only available from the final measurement location of the presumed tumor area while no histopathology was taken along the needle trajectory. However, all breast tumors were clearly visible on the US images and could confirm that the needle was positioned in normal tissue from the start of the measurements. Nevertheless, some uncertainty will still exist on the precise location of the tissue border where normal tissue ends and tumor tissue starts. It should furthermore be noted that the x-axes of the graphs in Fig. 5 are related to time opposed to distance, and thus these graphs therefore display a change over time. Since the needle was not moved with constant speed along the needle trajectory, it was not possible to display the measurements as a function of the distance.

In literature many different methodologies are used for classifying reflectance spectra of breast tissue, for example logistic regression [20, 22], classification and regression trees [35, 36], artificial neural network [24], hierarchical cluster analysis [24], k-nearest neighbor [37], linear discriminant analysis [35], and support vector machines [35, 38–41]. In this study, a linear SVM classifier was chosen, as this classifier is relatively insensitive to overfitting [42–46]. Possibly a polynomial kernel SVM would have provided better results, however because the number of patients in the study is limited for machine learning, a linear, less complicated, classifier was chosen. For similar reasons the bootstrap sampling was preferred to leave-one-patient-out cross-validation, even though bootstrap methods can have the tendency to be pessimistic. By extending the number of measurements the accuracy of the classification of the DRS measurements will likely improve, and more sophisticated machine learning algorithms can be used.

The SVM classification model was developed with the input of either fit parameters (fit parameter model), or all wavelengths in the spectrum (full spectrum model), or some selected wavelengths (selected wavelengths model). A previous publication comparing the classification accuracy for discrimination of breast cancer of a SVM model based on physical parameter data (equivalent to fit parameters model), with the accuracy of a SVM classification model based on empirical data (equivalent to the selected wavelengths model) reported similar results to this study [38]. The main advantage of using the fit parameters is that these parameters can provide insight into the physical and structural features that contribute to discrimination [38, 43]. However, if fit parameters cannot be estimated accurately, for example because the tissue has a layered structure, accuracy of classification models based on fit parameters will be lower [43]. This can explain why the accuracy of the fit parameter model was lower compared to the other two models.

We found that the performance of the selected wavelengths model is slightly better compared to the performance of the full spectrum model, which is not surprising since removing redundant wavelengths is often reported to be beneficial for classification performance [42]. There are many ways to select or reduce features, such as partial least squares [38], maximum representation and discrimination feature [20], or principal component analysis (PCA) [24, 38–40]. In this study, a Wilcoxon rank sum test is performed to find wavelengths that are significantly different between normal and tumor tissue. This method has been described for feature selection in previous publications, although in many cases this statistical test was preceded by PCA [40, 46]. The advantage of the Wilcoxon rank sum test is that the selection of wavelengths is based on true spectral differences between tissue types. The disadvantage is that wavelengths that are not discriminated according to this statistical test are excluded in the model development, although they could have discriminative power in combination with each other.

The wavelengths that were eventually selected are located in wavelength areas that are related to the absorption of light by fat, water, and to a lesser extent, blood. This result is in line with previous publications by others and our own group in which these substances also contributed to discriminating healthy tissue from tumor tissue [32, 38].

The DRS measurements in this study were obtained during breast biopsy procedures to provide a correlated dataset (DRS data and histopathology) and test the feasibility of real-time data acquisition. This setting is obviously different than the surgical setting where the goal is to classify DRS measurements of the resection margin. In that situation, the influence of air exposure will likely affect the visual wavelength range due to differences in oxygenated and de-oxygenated blood which have different optical absorption characteristics; whereas the near-infrared wavelength range, with predominant absorption characteristics from fat to water, will likely be less affected by the surgical setting. Furthermore, the resection margin can also be influenced by cauterization which was absent in the measurements obtained in the biopsy setting, or extravascular blood on the resection surface. With regard to the results in this study, this might imply that in the selected wavelengths model the first wavelength that was selected (501 nm) cannot be used. The accuracy of DRS measurements for the detection of tumor intra-operatively at the resection margin, should be investigated in a study in which DRS measurements (including also the NIR wavelengths) are acquired at the true resection margins, preferably in the surgical workflow.

Using DRS as a clinical margin assessment tool also requires that measurements can be acquired and classified in real-time. In the continuous dataset, each spectrum required 0.35 s to be acquired. If necessary this acquisition time could be decreased by a factor of 4 by increasing the fiber diameter from 200 to 400 μm. As for the classification this was not performed real-time in this study. However, once a classification model is defined the tissue can be classified in real-time as this requires little computational power.

Another important factor to consider is the influence of ambient light that might be different in the surgical resection field compared to the setup during a biopsy. Part of this challenge is overcome by the fact that a fiber is used which has to be in contact with the tissue instead of a non-contact configuration. Therefore, only light that falls in the acceptance angle of the fiber will be recorded by the spectrometer. However, in clinical practice this might mean that very bright light sources that are in close proximity of the fiber-optic probe have to be dimmed to ensure interference with the DRS measurements is prevented.

## Conclusions

In this paper, we demonstrate the feasibility that DRS measurements can be acquired real-time and that a predictive classification model can be built to classify the measurements as normal or tumor tissue. The classification model based on a selection of wavelengths discriminated normal tissue from tumor tissue with the highest accuracy and MCC of 0.93 and 0.87, respectively. This performance may be sufficient for the application of detecting positive resection margins during breast conserving surgery. The needle trajectory measurements show that DRS measurements can be acquired real-time and that these measurements can be classified accurately. Furthermore, the transition from normal tissue to tumor tissue was seen in the continuous DRS measurements.

Our current results indicate that integration of DRS in a surgical tool or knife could be useful for characterizing breast tissue in vivo and aiding surgeons in detecting positive resection margins during surgery. The next step is to investigate the feasibility of real-time DRS acquisition and classification on resection margins and investigate the impact of a DRS guided tool on surgical outcomes.

## Abbreviations

DRS: diffuse reflectance spectroscopy
SVM: support vector machine
MCC: Matthews Correlation Coefficient
SNV: standard normal variate
StO_2_: oxygen saturation
F/W-ratio: fat/water-ratio
PCA: principal component analysis
